# Conditional Regard in the Classroom: A Double-Edged Sword

**DOI:** 10.3389/fpsyg.2021.621046

**Published:** 2021-07-28

**Authors:** Yaniv Kanat-Maymon, Anat Shoshani, Guy Roth

**Affiliations:** ^1^Interdisciplinary Center Herzliya, Herzliya, Israel; ^2^Ben-Gurion University of the Negev, Beer Sheva, Israel

**Keywords:** conditional positive regard, conditional negative regard, engagement, self-determination theory, level of analysis

## Abstract

Teachers’ conditional positive and negative regard are widely endorsed teaching practices aimed to enhance students’ involvement and achievement in school. Previous research has mostly tapped the need frustration and harmful psychological well-being implications of these practices. Yet knowledge of their specific effects on school engagement is scant. This study investigated the association between students’ perceptions of homeroom teachers’ conditional positive and negative regard and their behavioral engagement, while considering the levels at which these practices are conceptualized and operate (a teacher characteristic and a student characteristic). Participants were *n* = 2533 students from 107 classes in the 7th to 10th grades. Multilevel analysis found conditional positive regard was positively associated with school engagement while conditional negative regard was inversely related. These findings were obtained at both the within- and between-class levels. Based on the findings, we argue conditional regard is a double-edged sword. Consistent with previous research, we suggest conditional negative regard has an undermining effect, and we point to conditional positive regard’s potential to enhance engagement. Lastly, we discuss the importance of the level of analysis and the alignment of theory with measurement.

## Introduction

School engagement is an area of concern for educators and researchers who are interested in promoting students’ positive academic experiences while minimizing their negative developmental outcomes. Approximately 5% of the population in the United States (2 million) and 10% in Europe (4 million) between the ages of 16 and 24 did not graduate from high school (European Commission, 2017; McFarland et al., 2020). Besides school dropout, involvement in delinquency, risky health behaviors, and aggression are other negative outcomes related to low school engagement (Greenwood et al., 2002; Fredricks et al., 2004). Positive outcomes associated with engagement include well-being and academic achievement (Fredricks et al., 2004; Upadyaya and Salmela-Aro, 2013).

School engagement may be of particular interest to educators, not only because it is related to a variety of important developmental outcomes but also because it is believed to be malleable (Sinclair et al., 2003). Indeed, research has shown engagement tends to decline as student age, especially in the transition to middle and high school (Klem and Connell, 2004; Wigfield et al., 2006), yet teachers’ and parents’ educational practices can bolster and sustain engagement over time (Fredricks et al., 2004; Nguyen et al., 2018).

One widely endorsed motivational practice considered to enhance students’ academic engagement and performance is conditional regard (Assor et al., 2004). Conditional regard involves the teacher’s or parent’s provision of affection and attention as dependent on the student’s attainment and enactment of desired academic expectations. Despite the popularity of this practice in the schooling context, in a study focused on school engagement, Assor et al. (2004) did not find a significant association between university students’ retrospective reports of parental conditional regard and school behavioral engagement. Moreover, research on conditional regard concludes this practice has pervasive negative consequences for children’s psychological well-being. For example, studies have reported conditional regard frustrates basic psychological needs (Moller et al., 2019), is related to psychological ill-being (Assor and Tal, 2012; Perrone et al., 2016; Wouters et al., 2018), and is associated with maladaptive motivation (Roth et al., 2009).

Given that conditional regard has negative consequences for youths’ psychological well-being and fails to produce the desired increase in school engagement (Assor et al., 2004; Roth et al., 2009; Cohen et al., 2019), it seems surprising that this practice is frequently used and widely endorsed by teachers and parents (Assor et al., 2004; Hoffmann et al., 2009). After all, increasing engagement and performance in school is the key utility of conditional regard.

To shed light on the association between conditional regard and engagement, we built on and extended Assor and colleagues’ work (2004) in three key ways. First, we focused on teacher-student relations. Research on student engagement has indicated that aside from factors related to the children themselves, the context within which learning take place shapes engagement (Fredricks et al., 2004; Nguyen et al., 2018). Sinclair et al. (2003) suggest parents’ and teachers’ educational approaches represent two major environmental influences that are particularly important to the formation of school engagement. Surprisingly, however, the majority of research on conditional regard has involved only parents; thus, little is known about the effects of teachers’ conditional regard. There are several noteworthy differences in the way teachers, as opposed to parents, influence student engagement. Teachers come in daily contact with their students, and they set the pace and goals of learning. Therefore, the impact of their teaching style on student engagement is both immediate and direct. Furthermore, unlike parents who mostly affect their own children’s engagement, teachers’ educational and disciplinary acts are likely to influence dozens of students each year. These aspects of the teacher’s role suggest the need for a more comprehensive understanding of the consequences of applying conditional regard in the classroom.

Second, we considered the recent distinction between two forms of conditional regard: *conditional negative regard* and *conditional positive regard* (Roth et al., 2009; Kanat-Maymon et al., 2015). Conditional negative regard involves providing less attention and affection than usual when the child does not enact desired behaviors; conditional positive regard involves providing more attention and affection than usual when a child enacts desired behaviors (Roth et al., 2009). While conditional negative regard is typically described as detrimental to well-being and motivation (Roth et al., 2009; Kaplan, 2018), conditional positive regard is viewed by some as less harmful and even benign due to its instantiation of affection and attention (Frost, 2005; McGraw, 2005).

Third, we considered the level of analysis at which conditional regard is generally conceptualized and analyzed. On the one hand, some scholars (Cohen et al., 2019) conceptualize conditional regard as a trait-like teaching style, reflecting a teacher’s global orientation toward enhancing motivation in the class; as such, it is expected to affect the entire class in the same manner (Heimlich and Norland, 2002). On the other hand, those endorsing a differential behavior approach to teaching (Babad, 2009) see conditional regard as a student-specific idiosyncratic phenomenon because the same teacher may use conditional regard differently with different students. According to this view, conditional regard may affect each student differently.

Based on these considerations, we differentiated between teachers’ positive and negative conditional regard and examined their unique associations with student behavioral engagement at both the individual and class levels.

## Hypotheses Development

### School Engagement

Typical classroom settings create frequent and sustained opportunities for behavioral engagement in learning. When students participate in activities, raise their hands in response to a question, pay attention to the teacher, or are actively involved in a reading or writing exercise, they are showing evidence of behavioral engagement. This type of behavioral engagement is a critical and common component of cognitive engagement (e.g., use of sophisticated learning strategies) and emotional engagement (e.g., positive emotional states, such as interest and enjoyment during a task). Although student engagement has been conceptualized as a multidimensional construct with behavioral, emotional, and cognitive aspects, behavioral engagement is more easily and directly observable in the classroom than emotional or cognitive engagement. As such, behavioral engagement is more often used by teachers to assess the effectiveness of their teaching style (Nguyen et al., 2018).

There is consensus that school children’s behavioral engagement is a key contributor to a wide range of positive educational outcomes (Greenwood et al., 2002; Fredricks et al., 2004). In the short term, studies suggest children’s engagement predicts learning, grades, and achievement test scores; in the long term, it predicts patterns of attendance, retention, graduation, and academic resilience (Jimerson et al., 2003; Sinclair et al., 2003; Skinner et al., 2009; Wang and Eccles, 2012; Lei et al., 2018). Moreover, work on classroom motivational dynamics considers behavioral engagement a key mediator between the teacher’s instruction and school outcomes (Skinner et al., 2009). Taken together, this body of work points to the usefulness of considering behavioral engagement as an outcome unto itself and gives credence to the goal of identifying antecedents in the teacher’s instruction that may foster or hinder students’ behavioral engagement (Greenwood et al., 2002; Skinner et al., 2009).

### Conditional Regard

Conditional regard is considered as a teaching practice by which teachers make their provision of affection and esteem contingent on students’ engagement and performance (Assor et al., 2004). Because students find the teacher’s affection and esteem to be important, it is commonly believed that granting or withholding regard can be used to direct students’ efforts to meet the teacher’s academic goals (Frost, 2005; McGraw, 2005). From an operant conditioning perspective, conditional regard represents the contingent administration of reinforcements and punishments, expected to increase the likelihood of desired behaviors such as putting effort into school tasks (McDowell, 1988; Gewirtz and Peláez-Nogueras, 1991). Similarly, theories of self-esteem, such as the sociometer theory of self-esteem (Leary et al., 1995) and contingencies of self-worth (Crocker and Park, 2004; Kanat-Maymon et al., 2019), suggest self-esteem has a motivating function. That is, people will be willing to put effort into specific domains in order to gain or maintain self-worth. This implies that a teacher’s conditional regard could motivate his or her students to be more engaged in school-related tasks.

Self-determination theory (SDT; Ryan and Deci, 2017) has a quite different view of the desirability of conditional regard. SDT postulates three basic psychological needs as causal mechanisms that energize and direct engagement: autonomy, competence, and relatedness (Reeve and Tseng, 2011). Autonomy is the need to self-regulate behavior in accordance with one’s sense of self, interests, and values (Ryan and Deci, 2017). Relatedness is the need to belong and feel socially connected to and cared for by others; in the schooling context, this would refer to teachers and classmates (Baumeister and Leary, 1995; Deci and Ryan, 2000). Competence is the need to develop personal capabilities and interact effectively with one’s environment, for example, to feel capable of completing an academic task (Deci and Ryan, 2000).

Several self-determination theory scholars have recently suggested conditional regard can be conceptualized as a need-thwarting practice, reflecting an inherent conflict or tension between the need for autonomy and relatedness (Kanat-Maymon et al., 2015; Kanat-Maymon et al., 2016; Cohen et al., 2019; Moller et al., 2019). In the school context, conditional regard requires students to trade or sacrifice some of their autonomy in exchange for some relatedness. That is, students are put in a situation where they are pressured to comply with the teacher’s achievement standards in order to gain or maintain the teacher’s approval.

According to self-determination theory, children’s autonomy and relatedness are equally important and essential for flourishing and well-being (Ryan and Deci, 2017). Because all needs are essential for high quality motivation, the provision of one (i.e., relatedness) may not compensate for the frustration of the other (autonomy) (Sheldon and Niemiec, 2006; Perreault et al., 2007). As an analogy, conditional regard is like watering a plant more frequently to compensate for a lack of sunlight – an excess of one element cannot make up for the absence of the other. The direct costs of basic psychological need frustration include loss of motivation and disengagement. For instance, using a three-wave longitudinal design, Jang et al. (2016) reported that an increase in high school students’ need frustration from the beginning of the school year to midway in the semester predicted a parallel increase in disengagement.

According to SDT, conditional regard is a need-thwarting practice and, as such, is expected to undermine engagement (Garn et al., 2018; Moller et al., 2019). To examine these relations, Assor et al. (2004) asked university students to recall maternal and paternal use of conditional regard in the academic domain during adolescence. Contrary to the researchers’ expectations, these parental perceptions were not correlated with the students’ reported behavioral engagement in school.

To elucidate the engagement consequences of conditional regard, subsequent studies refined the definition of conditional regard and differentiated between two forms: conditional negative regard and conditional positive regard (Roth et al., 2009). Conditional negative regard involves the withdrawal of affection and approval when a student does not meet the teacher’s expectations. The teacher can show disapproval by ignoring the student when he or she raises a hand, addressing the student in a cold, contemptuous, or impatient tone, and being less attentive, dismissing or criticizing the student’s ideas and opinions. Conditional positive regard involves providing more than usual levels of attention and affection when the student behaves as expected. To show approval, the teacher can allow the student more opportunities to speak, address him in a warm tone, listen more attentively, and address the student’s ideas and opinions carefully, positively, and considerately.

Cohen et al. (2019) and Roth et al. (2009) argued that conditional negative regard is more need-thwarting than conditional positive regard. Specifically, conditional negative regard requires sacrificing autonomy to avoid a decreased sense of relatedness. In the school context, it will thwart students’ relatedness because the teacher’s acceptance is likely to be withheld unless students meet certain academic demands. It may also undermine students’ sense of autonomy because it conveys to them that the teacher does not trust or believe they will volitionally meet expectations. In this scenario, the teacher forces the student to meet academic expectations by making affection contingent. Teachers’ conditional negative regard may therefore undermine not only students’ need for relatedness but also their need for autonomy. Indeed, Cohen et al. (2019) found a teacher’s conditional negative regard was negatively associated with students’ fulfilment of their autonomy and relatedness needs. Similarly, Garn et al. (2018) found students’ perceptions of teachers’ conditional negative regard were inversely associated with their sense of need satisfaction.

As conditional negative regard is experienced as need thwarting, it is likely to undermine engagement (Kanat-Maymon et al., 2012). In their self-system model of motivational development, Skinner et al. (2009) considered basic psychological needs to be intrapersonal resources that, when frustrated, de-energize engagement. Indeed, research on academic conditional negative regard has consistently found it correlates with school behavioral disengagement. For example, in a sample of 9th graders, Roth et al. (2009) found correlations between perceptions of academic parental conditional negative regard and disengagement. Kaplan (2018) found perceptions of teachers’ conditional negative regard were inversely correlated with school engagement in a sample of 10th to 12th grade children. Finally, in a sample of 7th to 10th grade children, Cohen et al. (2019) found teachers’ reports of conditional negative regard were negatively correlated with overall class ratings of agentic engagement (i.e., a proactive form of learning).

Conditional positive regard appears to be less harsh than the coercive and need-thwarting nature of conditional negative regard. In essence, conditional positive regard requires students to give up some of their autonomy and behave in ways they may not fully endorse in exchange for a greater sense of relatedness. That is, if students comply with the teacher’s expectations, they are guaranteed greater acceptance and appreciation than they normally receive. Hence, teachers’ conditional positive regard may thwart students’ need for autonomy but enhance their feelings of relatedness. Indeed, two studies found conditional positive regard was negatively correlated with fulfilment of the need for autonomy (Kanat-Maymon et al., 2015; Cohen et al., 2019). As for relatedness, a diary study among romantic partners found daily fluctuations in perceptions of a partner’s conditional positive regard were positively associated with daily fluctuations in relationship satisfaction, a proxy of relatedness (Kanat-Maymon et al., 2017). Interestingly, however, recent research by Cohen et al. (2019) found teachers’ conditional positive regard was not linked with students’ relatedness need satisfaction. While conditional positive regard seems to undermine the need for autonomy, it is less clear if it fully supports the need for relatedness. Given that conditional positive regard thwarts mostly autonomy and not relatedness, this practice may not be benign. At the same time, it may not be as coercive as conditional negative regard.

Mild exposure to need frustration may not necessarily diminish engagement; rather, it may trigger a change in the quality or locus of motivation (Vansteenkiste and Ryan, 2013). SDT holds a differential view of motivation, broadly categorized as autonomous vs. controlled. Autonomous motivation is characterized as based on interest, volition, and meaning. Controlled motivation is characterized as based on complying with forces alienated from the self, such as external contingencies (e.g., rewards and sanctions) and internal pressure (e.g., guilt and shame). According to SDT, need frustration may shift the locus of motivation from autonomous to controlled (Deci and Ryan, 2000).

Roth et al. (2009) argued that conditional positive regard often triggers a form of controlled motivation called introjected regulation. Introjected regulation refers to internal motivating forces that appeal to the individual’s feelings of guilt, shame, anxiety, and self-worth. Although such internal controls are psychologically maladaptive, in the school context, they may pressure students to adhere to the teacher’s expectations and thus secure student compliance (Soenens et al., 2012). Roth et al. (2009) found parental conditional positive regard was positively associated with grade-focused engagement, a measure of the extent to which a student is focused on attaining high grades. This association was mediated by a sense of internal compulsion (i.e., introjected motivation). Similarly, Assor and Tal (2012) found parental conditional positive regard was positively correlated with students’ compulsive over-investment in schoolwork. This construct refers to intense effort investment in schoolwork that is not experienced as fully volitional. Although a grade focus and compulsive over-investment are not direct measures of behavioral school engagement, to some extent, they grasp the notion of “working hard” on the school-related activities characterizing it.

Thus, although conditional positive regard may be experienced as distressing, it may foster behavioral engagement. Understanding the utility of this specific practice may also clarify its feasibility. After all, if conditional positive regard is such a harmful practice, why is this practice so frequently used and widely endorsed among teachers and parents? To some teachers, the utility of this practice in terms of school behavioral engagement may outweigh its psychological costs.

### Levels of Analysis

Levels of analysis issues have increasingly become a central focus of a significant volume of educational research specifying how teacher characteristics (i.e., class-level) contribute to the prediction of student-level outcomes (Lüdtke et al., 2009; Marsh et al., 2012). This reflects the increased recognition of the importance of aligning a theory with the data used to test it. Without proper alignment of theory and data, a theory cannot be subjected to valid testing, nor can valid conclusions be drawn from improperly aligned data or analyses (Gully and Phillips, 2019).

For example, the levels at which classroom conditional regard is expected to operate or the levels at which relationships are expected to hold are frequently not specified in research on conditional regard in the school context, and scholars often look at one level without considering the other (e.g., Kaplan, 2018). Ostroff (2019) argues associations that hold at one level of analysis may not hold at another or may even reverse their direction. Therefore, the hierarchical level at which conditional regard is exercised may be an important consideration in an examination of the nature of this practice and its impact on student engagement.

There is general consensus that conditional regard reflects a specific teaching style, namely a global orientation toward enhancing motivation (Heimlich and Norland, 2002; Assor et al., 2004). Accordingly, Cohen et al. (2019) conceptualized teachers’ conditional regard as reflecting a pervasive quality in the educational behavior of the teacher expected to persist across time, classes, and students. In other words, some teachers are more likely than others to use conditional regard to motivate their students. Therefore, all students in the same classroom are equally likely to be exposed to the teacher’s conditional regard. Hence, if the teacher’s conditional regard has any effect on student engagement, this should be evident in differences between classes, and analyses must be held at the class level, not at the individual student level (Marsh et al., 2012).

A common understanding of educational research is that teaching styles, such as conditional regard, are best captured by students’ reports (Kunter and Baumert, 2006; Wagner et al., 2016). Aggregation of the individual perceptions of the students in the same class may give a more accurate measure of the teacher’s use of conditional regard (Lüdtke et al., 2009). Of course, high agreement among students’ individual ratings of the teacher’s conditional regard is a prerequisite. In line with this approach, Cohen et al. (2019) aggregated student reports to produce class-level constructs and focused on how variability of teachers’ conditional regard was associated with between-class level variables.

Although teachers have a trait-like teaching style, they may use conditional regard differently with each of their students. Research on teachers’ differential behavior (Babad, 2009; Rubie-Davies, 2015) suggests teachers are guided by their beliefs about what students need and by their expectations about how students will respond if treated in particular ways. Teachers expect students to behave in specific ways and attain certain achievements. Thus, a teacher may use conditional regard differently with each student to motivate each one to achieve the teacher’s goals. If conditional regard can be differentially enacted by a teacher, each student will experience conditional regard in an idiosyncratic manner. Thus, if conditional regard is an idiosyncratic teacher-student experience, the student level is the appropriate level of conceptualization and analysis.

Garn et al. (2018) and Kaplan (2018) treated conditional regard as an idiosyncratic construct. Their analyses were performed at the individual student level using a path-analysis approach. However, this approach has limitations because it ignores the potentially conflated effect of the class level. An accumulating body of research on multilevel analysis and hierarchical data structure has clearly pointed out that ignoring higher-level variance can bias the estimation of lower-level parameters (Kozlowski and Klein, 2000; Kanat-Maymon et al., 2020). In nested data, class-level and student-level effects often conflate, making multilevel analysis a more appropriate approach as it can separate between-group and within-group effects. Therefore, in the present study, we applied a multilevel framework to estimate more precisely the effects of conditional regard at the class and student levels.

### Overview of the Study

The study examined two distinct aspects of teaching style, namely conditional negative regard and conditional positive regard, to determine their potential association with and relative contribution to students’ behavioral engagement at the class and student levels. Specifically, at the individual student level, we predicted students’ perception of their teacher’s conditional negative regard would be negatively linked with their behavioral school engagement whereas perception of their teacher’s conditional positive regard would be positively linked with their behavioral engagement. We made similar predictions at the class level. That is, classes who perceived their teacher as using conditional negative regard (aggregated score) would be inversely linked with class-level behavioral engagement, whereas classes who perceived their teacher as using conditional positive regard would be positively linked with class-level behavioral engagement.

## Materials and Methods

### Participants and Procedure

Participants were 2533 students from 107 classes at seven schools in central Israel. Students were asked to refer to their learning experience in classes taught by their homeroom teacher. Homeroom teachers in the Israeli educational system are responsible for helping students adjust to the school and maintain their well-being and are the main educational figures with whom students interact in the school. Homeroom teachers teach their homeroom class between 4 and 6 h weekly. Of the sample, 23% were 7th grade students (*n* = 594), 27% 8th grade students (*n* = 684), 21% 9th grade students (*n* = 530), and 29% 10th grade students (*n* = 725). Students’ ages ranged from 12 to 17 (*M* = 14.26, *SD* = 1.27), and 53% were female. Approximately 80% of the students reported their parents were married, and 70% reported average to very good economic status.

The institutional review board and the Israel Ministry of Education’s ethics committee approved this study. Written informed consent was obtained from both students and parents (i.e., active consent), and there were no objections to participating in the study. Students were assured their identity would remain confidential and anonymous. An identification coding system was used to ensure confidentiality. Participants were allowed to withdraw at any point, without having to provide a reason for doing so. The data collection started in the middle of the school year (March 2017). Trained research assistants (RAs) administered the questionnaires to students after teachers left the classroom. Students received further guidance on questionnaire completion via Qualtrics. When the session ended, the RAs explained the purpose of the study.

### Measures

All self-report measures were rated by students using a 5-point Likert scale ranging from 1 = *not at all* to 5 = *very much*.

#### Perceived Teacher Conditional Positive and Negative Regard

Perceived teachers’ conditional regard was assessed using a modified version of the 10-item parental academic conditional regard scale (Roth et al., 2009; Assor and Tal, 2012). The original scale assesses the extent to which participants perceive their parents as using conditional positive and negative regard upon their fulfillment of parental expectations of academic engagement and achievement. The scale was modified to assess the extent to which students perceived their homeroom teachers as using conditional regard in the classroom. The modified 5-item conditional positive regard included such items as “When I study hard, I feel that my teacher appreciates me much more than usual,” and the modified 5-item conditional negative regard included such items as “When I do not succeed at class, my teacher shows me less caring and attention than usual.”

To support the psychometric properties of the modified perceived teacher scale, the 10 items were subjected to confirmatory factor analysis (CFA). CFA results revealed the two-factor model had good fit indices, χ^2^(34) = 409.72, *p* < 0.001, NFI = 0.96, CFI = 0.96, TLI = 0.94, and RMSEA = 0.06, with all items loading on their intended latent factor, ranging from 0.41 to 0.79. Correlation between factors was moderate, *r* = 0.54, *p* < 0.001. This two-factor model fit the data better than a one-factor model in which all items loaded on a single latent factor, χ^2^(35) = 2353.54, *p* < 0.001, NFI = 0.75, CFI = 0.75, TLI = 0.61, RMSEA = 0.16, Δχ^2^(1) = 1943.82, *p* < 0.001. Cronbach’s alpha were 0.86 for conditional positive regard and 0.78 for conditional negative regard.

#### Behavioral Engagement

Behavioral engagement was assessed using four items from Skinner et al. (2009) Engagement vs. Disaffection with Learning measure. The items tapped students’ effort (“In this class, I work as hard as I can”), attentiveness (“I pay attention in class”), class participation (“I participate in class discussions”), and completing assignments (“I complete my class assignments”). Cronbach’s alpha was 0.71.

#### Control Variables

Given theory and research suggesting students’ engagement decreases as they grow older (Wang and Eccles, 2012), we used grade level as a control variable. Student and teacher gender were also controlled for, in light of theories on gender socialization, suggesting autonomy is less important for females (Helgeson, 1994) and relatedness is less important for males (Gilligan, 1982). Lastly, although the small school sample size (*N*_school_ = 7) was insufficient to include it as a third level in the multilevel model, it was important to account for its potential covariance.

### Measurement Model

To examine the psychometric properties of the conditional regard and engagement measures, all relevant items were subjected to CFA. We constrained each item in the measurement model to load on the factor it was designed to estimate. We did not correlate residual terms for the items. In addition, we did not impose equality constraints on factor loadings, and factor covariances were free to be estimated. Results revealed the three-factor solution fit the data well, χ^2^(74) = 637.86, *p* < 0.001, NFI = 0.95, CFI = 0.95, TLI = 0.93, RMSEA = 0.06. All items had significant loadings (ranging from 0.40 to 0.79, *p* < 0.001) on their intended latent factor.

### Analytical Strategy

As the study was conducted in a school setting, where students are nested in classes and schools, we applied a multilevel modeling approach using the SPSS mixed procedure (SPSS, 2005). First, we partitioned the variance in the dependent variable, behavioral engagement, into its student-level, class-level, and school-level components. The variance between schools (i.e., Level 3) accounted for less than 0.3% of the overall variance in engagement; therefore, we retained a two-level model, nesting students within classes. Students’ variability in engagement (σ^2^ = 0.71) accounted for 94% of the overall variance, and class-level variability (τ = 0.04) accounted for the remainder (ICC = 0.06). Heck et al. (2013) suggest a multilevel model is warranted if the ICC is higher than 0.05.

To avoid conflating students’ and classes’ levels of conditional regard, we decomposed the variance in conditional regard into within- and between-class components. Within-class conditional positive and negative regard were group-centered (students’ deviation from class mean), and class means (aggregated scores) for conditional regard were introduced at the class level (Kozlowski and Klein, 2000). To ease coefficient interpretation, class means were grand-centered (classes’ deviation from the grand mean). Student-level intercepts and slopes were treated as random effects (i.e., a random intercept and slope model). Gender and grade levels were included as covariates to account for their potential effects.

### Aggregation Test

To justify the appropriateness of aggregating students’ ratings of perceived conditional regard as reflecting a teaching style, it is necessary to demonstrate that students are in agreement (e.g., students within a class have a similar experience of their teacher’s conditional regard). To this end, we assessed both within-class agreement and between-class variability (Bliese, 2000). First, we calculated ICC1. In general, ICC1 may be interpreted as the proportion of variance in students’ ratings that is attributed to systematic between-class differences compared to the total variance in ratings. Values as small as 0.05 may provide *prima facie* evidence of a small to medium group effect size (LeBreton and Senter, 2008). ICC1 values were significant for both conditional positive regard (ICC1 = 0.05, *p* < 0.05) and conditional negative regard (ICC1 = 0.06, *p* < 0.05). Next, we calculated ICC2 to determine the reliability of class means. ICC2 estimates the stability (i.e., reliability) of mean ratings from different students (i.e., judges) for different teachers (i.e., targets). The ICC2 for conditional positive regard was 0.54 and for conditional negative regard 0.60, indicating moderate agreement (LeBreton and Senter, 2008). We also calculated the within-group agreement (rwg; James et al., 1993) of the conditional regard measures to assess whether the class members were uniform in their teacher ratings to such an extent that perceptions could be perceived as shared. Rwg was 0.86 for conditional positive regard and 0.86 for conditional negative regard and thus reached the required minimum (rwg > 0.70; LeBreton and Senter, 2008). Finally, results of a one-way random ANOVA indicated significant between-class differences for both conditional positive regard, F(106, 2414) = 2.19, *p* < 0.001, and conditional negative regard, *F*(106, 2414) = 2.53, *p* < 0.001. In sum, aggregating conditional regard to a class-level construct was empirically justified.

## Results

Table 1 presents descriptive statistics and correlations for variables at both within- and between-class levels. At the within-class level, perceived conditional positive regard and perceived conditional negative regard were positively associated with engagement. At the between-class level, perceived conditional positive regard was positively associated with engagement but perceived conditional negative regard was not. These correlations should be interpreted with caution, as they do not account for the covariance between the two forms of conditional regard at both the within- and between-class levels.

**TABLE 1:** Descriptive statistics and correlations between the research variables.

	M	SD	1	2	3	4
	**Within-level (*n* = 2526)**					
1. Engagement	3.59	0.87				
2. CPR	2.61	1.05	0.21***			
3. CNR	2.02	0.88	–0.01	0.45***		
4. Gender	–	–	−0.05*	–0.05	−0.10**	
	**Between-level (*n* = 107)**					
1. Engagement	3.59	0.28				
2. CPR	2.61	0.33	0.21*			
3. CNR	2.02	0.29	–0.08	0.57***		
4. Grade level	8.63	1.12	−0.25**	−0.27**	–0.18	
5. Gender (% girls)	52.90	16.49	–0.06	−0.29**	−0.33***	0.26**

CPR, perceived teacher’s conditional positive regard; CNR, perceived teacher’s conditional negative regard. Gender is coded as 1 = male, 2 = female. Grade level refers to 7th to 10th grades. *p < 0.05, **p < 0.01, ***p < 0.001.

Table 2 presents the unique statistical effects of conditional positive regard and conditional negative regard as predictors of behavioral engagement. As shown in the upper part of the table, conditional positive regard was positively associated with engagement. This finding suggests students who thought the teacher used more conditional positive regard (in comparison to other students) were also more behaviorally engaged in class work. In contrast, conditional negative regard was inversely associated with engagement, whereby students who experienced the teacher as using more conditional negative regard (in comparison to other students) were less engaged in class work. We also noticed a gender effect: girls were more engaged than boys. A comparison of the within-class residual variances (σ^2^) prior to and following the introduction of the predicting variables indicated that the within-class model accounted for 13% of the variance in student engagement.

**TABLE 2:** Conditional positive and negative regard as predictors of behavioral engagement: Unstandardized within-class and between-class coefficients.

	B	SE	*t*	*p*	[95%CI]
**Within-class effects**					
Intercept	3.48	0.04	78.25	< 0.001	[3.35, 5.59]
CPR	0.24	0.02	11.61	< 0.001	[0.20, 0.28]
CNR	–0.14	0.02	5.76	< 0.001	[−0.19, −0.09]
Gender (girls)	0.10	0.03	2.89	0.004	[0.03, 0.16]
**Between-class effects**					
CPR	0.37	0.09	3.97	< 0.001	[0.18, 0.55]
CNR	–0.34	0.10	3.34	< 0.001	[−0.55, −0.14]
Gender (% girls)	–0.00	0.00	0.17	0.862	[−0.00, 0.00]
Grade level	–0.03	0.02	1.62	0.109	[−0.08, 0.01]

*CPR, perceived teacher’s conditional positive regard; CNR, perceived teacher’s conditional negative regard. For gender, boys are the reference group. Random coefficients are σ^2^ = 0.62 and τ = 0.03.*

At the between-class level (bottom part of Table 2), conditional positive regard was positively associated with engagement. This finding suggests that in classes where teachers were using more conditional positive regard (in comparison to other classes), average class engagement was higher. Conditional negative regard was inversely associated with engagement. That is, in classes where teachers were perceived as using more conditional negative regard (in comparison to other classes), average class engagement decreased. Gender and grade level were not associated with engagement. A comparison of between-class residual variances (τ) prior to and following the introduction of the predicting variables showed that the between-class model accounted for 24% of the variance between classes’ engagement.

To examine the findings’ robustness, we conducted three further sets of analyses. First, to account for school effects, we replicated the analysis using a three-level model in which students were nested within classes and classes nested within schools. Results of the three-level model did not diverge from those of the two-level model. Second, we included gender and age as moderators. Results indicated a significant within-class level gender by conditional negative regard interaction (B = −0.11, SE = 0.04, t = 2.30, *p* = 0.022). When we probed the interaction, we discovered the negative association of conditional regard with engagement was stronger for girls (B = −0.19, SE = 0.04, t = 2.25, *p* = 0.024) than for boys (B = −0.09, SE = 0.04, t = 4.75, p < 0.001). We did not find a significant within-class level gender by conditional positive regard interaction (B = −0.06, SE = 0.04, t = 1.70, *p* = 0.090). At the between-class level, conditional positive and negative regard were not moderated by gender (CPR: B = 0.001, SE = 0.007, t = 0.12, *p* = 0.906; CNR: B = −0.001, SE = 0.008, t = 0.86, *p* = 0.390) or grade level (CPR: B = −0.03, SE = 0.08, t = 0.41, *p* = 0.686; CNR: B = 0.15, SE = 0.10, t = 1.47, *p* = 0.145).

Third, we repeated the analysis without controlling for gender and grade level. This is in line with Simmons et al. (2011) claim that comparing with and without covariates analyses allows non-biased transparency which is the extent that a finding relies on the presence of a covariate. Results showed our main findings were not altered by the exclusion of gender and grade level.

## Discussion

The goal of the study was to explore the correlations between teachers’ conditional positive and negative regard and students’ behavioral engagement in the classroom. Based on previous research involving teachers’ and parents’ conditional regard, we hypothesized that perceptions of teachers’ conditional negative regard would be negatively linked with students’ engagement, while teachers’ conditional positive regard would have a positive association. Unique to this research was the attempt to explore conditional regard both as a teaching style or teacher characteristic expected to have an environmental effect and predict engagement variance between classes and as a teacher’s differential behavior expected to predict variance in engagement within classes.

The findings revealed unique statistical effects for perceived conditional positive regard and conditional negative regard in predicting student engagement. Note that the effects of each form of conditional regard were obtained while controlling the variance of the other form of conditional regard. The multilevel approach detangled within-class from between-class effects; it revealed that, as expected, students who perceived their teacher as using more conditional positive regard in the classroom were more engaged in class work. Similarly, classes who perceived the teacher as using more conditional positive regard showed higher average engagement. In contrast, students who perceived the teacher as using conditional negative regard were less engaged in classroom work, while conditional positive regard shared variance partialed out. This negative association was more evident for girls than boys. At the between-class level, while controlling for conditional positive regard, classes who, on average, perceived the teacher as using more conditional negative regard were, on average, less engaged.

Our finding of a negative association between conditional negative regard and engagement is in line with previous research on teachers’ and parents’ conditional regard. For instance, Roth et al. (2009) found perceived academic parental conditional negative regard was associated with school children’s disengagement, and Kaplan (2018) found perceived teacher conditional negative regard was negatively correlated with students’ engagement. Research grounded in self-determination theory attributes the disengagement related to conditional negative regard to its thwarting of the basic human needs for autonomy and relatedness (Cohen et al., 2019). The meeting or thwarting of basic needs is understood as a subjective experience that energizes behaviors and effort. When needs are seriously thwarted, disengagement results (Reeve and Tseng, 2011).

The results indicated conditional negative regard was more strongly associated with disengagement among girls than boys. Assor and Shavit-Miller (2012) argued girls are more vulnerable to conditional negative regard because of early gender socialization when girls’ sense of worth is more heavily dependent on the satisfaction of their relatedness needs. Consequently, girls may find it more difficult to tolerate parental love withdrawal. In a qualitative study, Assor and Shavit-Miller (2012) also examined an alternative explanation accordingly girls show hyper sensitivity to negative events in general. In this study, girls who were vulnerable to conditional negative regard were not more sensitive than boys to signs of frustration stemming from social influence and achievement needs. Assor and Shavit-Miller (2012) concluded that girls vulnerability to conditional negative regard is attributed to their sensitivity to relatedness need frustration and not a result of sensitivity to negative events in general.

Our study also demonstrates that the negative association between conditional negative regard and behavioral engagement is not susceptible to a conflated effect characterizing a nested data structure (Kaplan, 2018). By decomposing conditional regard to its within- and between-class levels, we could account for teachers’ variances in conditional negative regard when estimating the within-class effects. When added to previous findings on the implications of conditional negative regard for basic need satisfaction and engagement, our findings contribute to the emerging conceptualization of a teacher’s conditional negative regard as a controlling practice that undermines student engagement (Bartholomew et al., 2018; Garn et al., 2018).

Although our MLM results suggest negative correlations between conditional negative regard and engagement, these results should be interpreted with caution, as the zero order correlations were not statistically significant. The finding may suggest the emergence of a suppression effect in the data. In suppression, the assessment of a predictor includes an additional variance that blears or suppresses the predictor-criterion correlations. Inclusion of a direct measure of the disruptive variance in regression-based analysis removes the irrelevant variance in the predictor and thus indirectly allows a more concise estimate of the predictor-criterion relationship. Conditional positive regard and conditional negative regard are positively correlated. If they associate with engagement in opposite directions, the positive associations between conditional positive regard and engagement may suppress the negative associations between conditional negative regard and engagement, resulting in low magnitude Pearson correlations. However, in the MLM, when the variance of conditional positive regard is statistically removed from the data, the negative correlations of conditional negative regard can more clearly appear. Although cumulative research, including our study, implies that conditional negative regard undermines student engagement, further research is needed to establish valid conclusions.

As hypothesized, conditional positive regard was correlated with high engagement at both the within-and between-class levels. To the best of our understanding, our study is the first to discover a direct relationship between conditional positive regard and student engagement. Previous research, including work by Roth et al. (2009) or Assor and Tal (2012), found conditional positive regard was correlated with intense preoccupation with school performance. However, this research did not directly tap the concept of school engagement, by which we mean working hard in class. In fact, research on goal achievement suggests preoccupation with high grades does not necessarily lead to engagement; it sometimes leads to alternative paths such as cheating (Kanat-Maymon et al., 2015).

Many see conditional positive regard as a relatively benign disciplinary practice, as it entails additional provision of affection and acceptance when the child meets the teacher’s or the parent’s expectations. Thus, practices similar to conditional positive regard are often recommended in popular education and parenting guides (e.g., Steinberg, 2004; McGraw, 2005). The positive association we found between conditional positive regard and school engagement at both the student and class levels may shed light on the value of this practice. Our findings suggest that, unlike conditional negative regard, conditional positive regard can be viewed as an engagement-enhancing teaching practice.

However, it is worth noting that conditional positive regard is not all that benign, and these teaching practices come at a cost. Research indicates conditional positive regard frustrates the basic need for autonomy while not fully satisfying the need for relatedness (Kanat-Maymon et al., 2017; Cohen et al., 2019). The evolving body of research on need frustration (Vansteenkiste and Ryan, 2013) suggests mild frustration does not automatically lead to disengagement, but it may trigger less adaptive forms of coping. One common implication of autonomy need frustration is the adoption of a controlled motivation (Bartholomew et al., 2018), a motivating style in which behavior is experienced as alienated from the self and often accompanied by contingent self-worth and distress (Ryan and Deci, 2017). Not surprisingly, then, research on parental conditional regard has found this practice is correlated with controlled motivation (Roth et al., 2009), fluctuations in self-esteem (Otterpohl et al., 2019), and anxiety (Wouters et al., 2018). Therefore, the adoption of this practice as a means to enhance student engagement is not an easy decision for educators.

Our research results also shed light on the level at which conditional regard operates. Our decomposition of the variance in conditional regard revealed conditional regard can be conceptualized as a teaching style that may differ between classes and, at the same time, be conceptualized as an idiosyncratic practice, whereby a teacher can use conditional regard differently with each student. Interestingly, conditional regard had similar effects on engagement at both the student and class levels. However, the fact that conditional regard operates at these two levels does not undermine the importance of aligning theoretical conceptualizations with measurement. Much has been written about the importance of the level at which a variable operates (Gully and Phillips, 2019), and many studies have demonstrated that the same variable can have an effect at one level and not have an effect or even have an opposite effect at another level (Ostroff, 2019). Conditional regard is no exception. We call on researchers who define conditional regard as a phenomenon that characterizes the learning environment to focus on the consequences of conditional regard at the interclass level; that is, how a group of students studying in an environment of high conditional regard differs from a group of students studying in an environment characterized by low conditional regard. In contrast, researchers who treat conditional regard as a phenomenon occurring between the teacher and the specific student should focus on the implications of conditional regard, looking at individual students’ functioning while controlling for intergroup effects.

Overall, our study highlights the importance of detangling the variability that results from differences between students from the variability that results from systematic differences between classes or teachers. The findings demonstrate that a substantial part of the variance in both engagement and conditional regard is at the student level. Thus, at first glance, it may seem the effects of conditional regard at the class level have a negligible effect on student engagement when compared to effects at the student level. However, statistically small effects can have a wide impact under two conditions: if they apply to many students or if they apply repeatedly to the same student. When combined, even small effect sizes can have a substantial impact. Given that the student-teacher ratio in secondary schools ranges from 1:20 to 1:30 students per teacher, and as students are exposed to the teacher throughout the whole academic year, even small between-class effects might have a broad impact on individual students.

The limited amount of variance in the between-class level of engagement relative to the within-class level could have resulted from the procedure we used to create the between-class variables. To arrive at the between-class measures of engagement and conditional regard, we aggregated students’ individual ratings to the class level. Individual-level data are more susceptible to individual difference bias, and this might inflate the within-class variance. Of course, teachers’ reports can be used instead of aggregated students’ reports. However, even this approach has been criticized, as it is not clear that teachers are the best source of information on their own teaching style (Bardach et al., 2018). Future research should include both students’ reports and teachers’ reports and replicate the findings across reporters.

A few important limitations should be considered when interpreting the results of the study. First, a cross-sectional design precludes causal conclusions and raises the possibility of alternative interpretations (Matos et al., 2018). Further longitudinal research is needed to shed light on the causal impact of teachers’ conditional regard. Second, the study focused on the behavioral aspect of student engagement. Given that engagement is a multidimensional construct (Skinner et al., 2009), it is too early to generalize the findings to other dimensions, such as emotional and cognitive engagement. Third, the study used students’ self-reports to estimate teachers’ conditional regard and their own engagement. Although students’ reports are acceptable and have been found reliable in tapping teachers’ behaviors, future studies should add other forms of data, such as teachers’ reports. Fourth, we did not examine the aspects of students’ basic psychological needs in the context of their academic performance in the class, and such an examination could broaden the understanding of the dynamics of conditional regard.

## Data Availability Statement

The raw data supporting the conclusions of this article will be made available by the authors, without undue reservation.

## Ethics Statement

The studies involving human participants were reviewed and approved by The institutional review board and the Israel Ministry of Education’s Ethics Committee. Written informed consent to participate in this study was provided by the participants’ legal guardian/next of kin.

## Author Contributions

YK-M conceived of the study, participated in the design, data collection, analysis for the study, and drafted the manuscript. AS and GR participated in its design, data collection, and contributed to drafts of the manuscript. All authors read and approved the final manuscript.

## Conflict of Interest

The authors declare that the research was conducted in the absence of any commercial or financial relationships that could be construed as a potential conflict of interest.

## Publisher’s Note

All claims expressed in this article are solely those of the authors and do not necessarily represent those of their affiliated organizations, or those of the publisher, the editors and the reviewers. Any product that may be evaluated in this article, or claim that may be made by its manufacturer, is not guaranteed or endorsed by the publisher.
